# A 0.8 V, 5.3–5.9 GHz Sub-Sampling PLL with 196.5 fs_*rms*_ Integrated Jitter and −251.6 dB FoM

**DOI:** 10.3390/s21227648

**Published:** 2021-11-18

**Authors:** Shi Zuo, Jianzhong Zhao, Yumei Zhou

**Affiliations:** 1 Smart Sensing R & D Centre, Institute of Microelectronics of Chinese Academy of Sciences, Beijing 100029, China; zuoshi@ime.ac.cn (S.Z.); zhaojianzhong@ime.ac.cn (J.Z.); 2 Institute of Microelectronics, University of Chinese Academy of Sciences, Beijing 100049, China

**Keywords:** SSPLL, hybrid dual path loop, low jitter, low power consumption

## Abstract

This paper proposes a hybrid dual path sub-sampling phase-locked loop (SSPLL), including a proportional path (P-path) and an integral path (I-path), with 0.8 V supply voltage. A differential master–slave sampling filter (MSSF), replacing the sub-sampling charge pump (SSCP), composed the P-path to avoid the degraded feature caused by the decreasing of the supply voltage. The I-path is built by a rail-to-rail SSCP to suppress the phase noise of the voltage-controlled oscillator (VCO) and avoid the trouble of locking at the non-zero phase offset (as in type-I PLL). The proposed design is implemented in a 40-nm CMOS process. The measured output frequency range is from 5.3 to 5.9 GHz with 196.5 fs root mean square (RMS) integrated jitter and −251.6 dB FoM.

## 1. Introduction

Since 1969, the LC-based phase-locked loops (PLLs) have been developed for over fifty years [1]. Several designs of LC-based PLLs are reported recently to save power consumption [2,3,4,5,6,7,8] as urgent demands of low power requirements for integrated circuits (ICs) appear. Decreasing supply voltage is an effective way to achieve low power. However, low voltage always limits the performance of circuits. Therefore, improving the noise feature with lower supply voltage is very attractive.

In a classical charge pump PLL (CPPLL) system [9,10,11,12,13,14,15], phase noise is mainly generated from two parts: out-of-band noise which is dominated by the voltage-controlled oscillator (VCO) (noise of low pass filter is neglected); and in-band noise which is dominated by the phase detector, charge pump and divider. Several efforts have been addressed to study the phase noise of VCOs [16,17,18] and designs with ultra-low power consumption have been published [19,20,21]. Hence, this paper concentrates on the improvement of in-band noise at low supply voltage.

From the literature review, compared with widely used CPPLLs, sub-sampling PLL (SSPLL) and type-I PLL can suppress in-band noise effectively. In an SSPLL system, the multiplication factor N of the noise of charge pump is rejected and the noise of divider is removed [22,23,24]. However, the sub-sampling charge pump (SSCP) is not suitable for low voltage application, since it suffers from deteriorated current noise. In a type-I PLL, although the exclusive-OR gate and the master–slave sampling filter (MSSF) can work well with low voltage [25], the suppression of out-band noise is limited. Furthermore, locking at non-zero phase offset which causes spur noise is a serious trouble for type-I PLL. Therefore, the architectures of SSPLL and type-I PLL cannot be utilized for low voltage application directly.

Some previous published low-voltage PLL designs [2,3,4,5] demonstrate that transferring the PLL loop into a dual path loop which includes a proportional path (P-path) and an integral path (I-path) is a beneficial method to mitigate the limitation of circuits performances at the architectural level. Therefore, the dual path system might provide a chance for designers to take full advantage of the SSPLL and type-I PLL and, meanwhile, avoid the drawbacks of these two structures.

Based on the problems and the possible idea of the solution, we present a hybrid dual path type-II SSPLL which can work at 0.8 V supply voltage with 196.5 fs${}_{rms}$ integrated and −251.6 dB FoM (figure of merit as expressed in Equation (1) [26]).

(1) $$FoM_{PLL}=20\log\left(\frac{Jitter_{rms}}{1\;s}\right)+10\log\left(\frac{Power}{1\;\mathrm{mW}}\right)$$

## 2. Architecture of Proposed SSPLL

### 2.1. Conceptual Block Diagram

**Figure 1:**
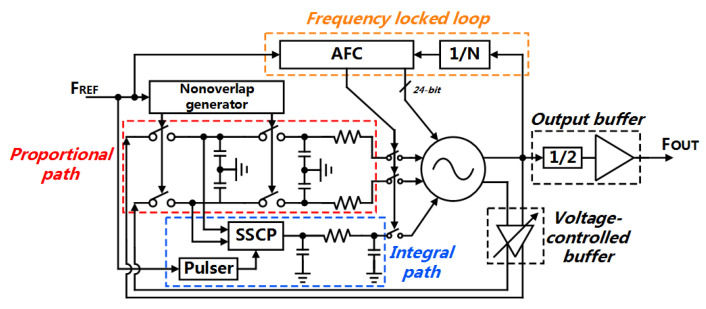
Conceptual architecture of proposed hybrid dual path SSPLL.

The main objective of this study is to design a dual path type-II SSPLL to reduce the in-band noise with low supply voltage. As mentioned above, the MSSF, in the type-I PLL, can operate well at low voltage without feature limitation. Hence, the MSSF can take charge of the P-path directly. The SSCP is kept to compose the I-path, in order to suppress the noise of the VCO by the type-II structure. Although the performance of the charge pump is degraded, the gain of the I-path is much smaller than the gain of the P-path, therefore, the noise contribution in the PLL system is much smaller than that of the P-path (the theoretical verification will be implemented in the following sections). Moreover, the sub-sampling-based PLL has the probability to lock at an unwanted frequency, so a frequency-locked loop (FLL) is necessary to be added to avoid this frequency uncertainty. We design an all-digital FLL (ADFLL) which consists of a digital divider and an adaptive frequency calibration (AFC) [27]. The all-digital circuit is beneficial for the power saving as it can be fully powered off, after the calibration is done. The overall conceptual block diagram for the proposed system is presented in Figure 1.

As indicated in [25], the traditional exclusive-OR based type-I PLL always faces the spur noise trouble, because of locking at the non-zero phase offset. Although the ideal MSSF can remove the voltage ripple on the control line of the VCO, current leakage from varactors and clock feedthroughs from the MSSF would introduce large ripples. To mitigate this phenomenon, we propose a differential MSSF to transfer the single path ripples into common-mode ripples.

Furthermore, we add a voltage-controlled buffer (VCBUF) at the output of the VCO to isolate the signal of the VCO away from the changeable load from the MSSF during the sampling.

### 2.2. Loop Analysis

**Figure 2:**
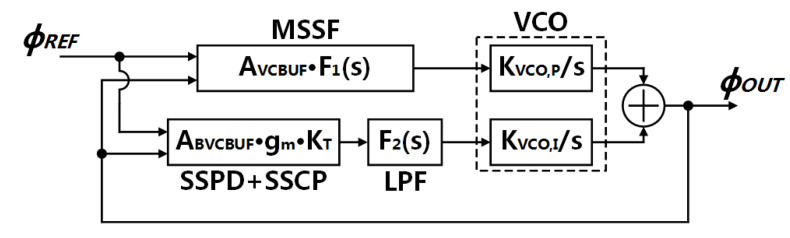
Linear model of the proposed SSPLL.

A linear phase-domain model of the proposed SSPLL system is shown in Figure 2. We can treat this model as a time-continuous system if the bandwidth (BW) of the PLL $f_{BW}$ is an order of magnitude smaller than that of the reference clock frequency $f_{REF}$ [28] (called “Gardner’s Limit”).

Unlike the traditional SSPLL, the proposed system has two paths as mentioned above. The relationship of these two paths determines the stability of the loop and phase noise performance of the system. Hence, we need to analyze the transfer function first.

**Figure 3:**
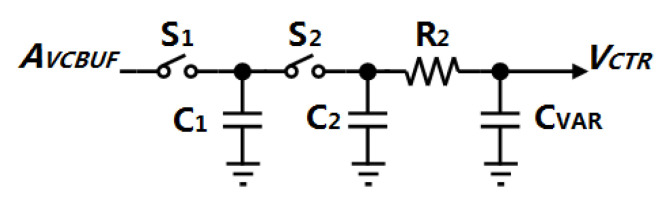
Single-end structure MSSF.

In the P-path, to simplify the calculation, a single-end structure of the MSSF is analyzed in Figure 3, where $C_{VAR}$ is the capacitance of the varactor in the VCO.

As a continuous-time approximation, since the Gardner’s Limit is assumed to be satisfied, the switched capacitor $C_{1}$ can act as a series-equivalent resistor $R_{1}$:

(2) $$R_{1}=\frac{1}{f_{REF}\cdot C_{1}}$$

where $f_{REF}$ is the reference clock frequency. The gain of the MSSF in s-domain is given as Equation (3).

(3) $$F_{1}\left(s\right)=\frac{sR_{2}C_{VAR}+1}{s^{3}R_{1}R_{2}^{2}C_{2}C_{VAR}^{2}+s^{2}R_{2}C_{VAR}\left(2R_{1}C_{2}+R_{1}C_{VAR}+R_{2}C_{VAR}\right)+s\left(R_{1}C_{2}+R_{1}C_{VAR}+2R_{2}C_{VAR}\right)+1}$$

where we choose $C_{1}$ = 64 fF, $C_{2}$ = 16 fF, $C_{VAR}$ = 10 fF and $R_{2}$ = 21 kΩ to push the poles and zeros far away from $f_{BW}$ and the gain of the MSSF can be approximated as 1. Thus, the open-loop gain of the P-path is obtained as follows:

(4) $$G_{PP}\left(s\right)\approx A_{VCBUF}\cdot 1\cdot \frac{K_{VCO,P}}{s}$$

where $A_{VCBUF}$ is donated as the amplitude of the output signal from the voltage-controlled buffer.

Then, in the I-path, we can get the gain of the SSCP directly from the introduced modeling in the traditional SSPLL [22]:

(5) $$\beta_{SSCP}=A_{VCBUF}\cdot g_{m}\cdot \frac{\tau_{PUL}}{T_{REF}}=A_{VCBUF}\cdot g_{m}\cdot K_{T}$$

where $g_{m}$ is the transconductance of the input transistor of the SSCP, $\tau_{PUL}$ is the pulse width of the pulser, $T_{REF}$ is the period of the reference clock. Moreover, the transfer function of the I-path low pass filter (LPF) can be easily acquired:

(6) $$F_{2}\left(s\right)=\frac{sR_{int}C_{int2}+1}{s^{3}R_{int}^{2}C_{int1}C_{int2}^{2}+s^{2}R_{int}C_{int2}\left(2C_{int1}+C_{int2}\right)+s\left(C_{int1}+C_{int2}\right)}$$

where $C_{int1}$ is the integral capacitor, and $R_{int}$ and $C_{int2}$ are the low pass resistor and capacitor, respectively. We choose $C_{int1}\gg C_{int2}$ and 1/$2\pi R_{int}C_{int2}\gg f_{BW}$ to get $F_{2}\left(s\right)\approx 1/sC_{int1}$. Hence, the open-loop gain of the I-path is given as:

(7) $$G_{IP}\left(s\right)\approx A_{VCBUF}\cdot g_{m}\cdot K_{T}\cdot \frac{K_{VCO,I}}{s^{2}C_{int1}}.$$

If we set $K_{VCO,P}=K_{VCO,I}=K_{VCO}$, the system open-loop gain is:

(8) $$G_{SSPLL}\left(s\right)=G_{PP}\left(s\right)+G_{IP}\left(s\right)=\frac{A_{VCBUF}\cdot K_{VCO}}{s}\cdot \left(1+\frac{g_{m}K_{T}}{sC_{int}}\right)$$

where a zero appears at $f_{z}=g_{m}K_{T}/2\pi C_{int1}$.

In order to ensure the stability of the SSPLL, the zero needs to be smaller than $f_{BW}$. In other words, $g_{m}K_{T}$ should be much smaller than $sC_{int1}$ around the angular frequency of the reference $\omega_{BW}$, which means that $G_{PP}\left(s\right)\gg G_{IP}\left(s\right)$. Hence, $f_{BW}$ is determined by the P-path.

## 3. Circuit Implementation

### 3.1. Class-C VCO with Start-Up Circuit

**Figure 4:**
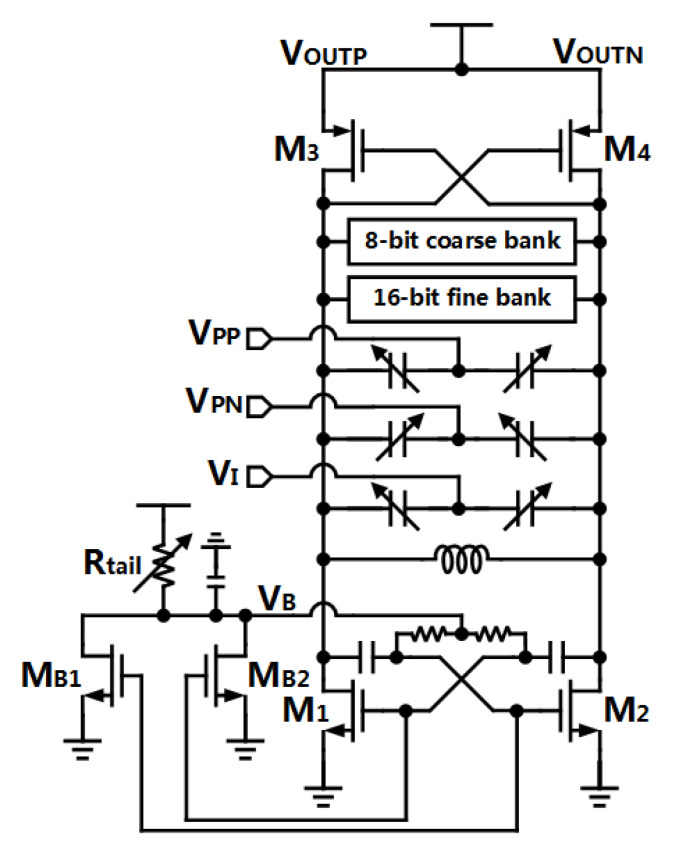
Structure of the proposed complementary cross-coupled Class-C VCO.

Due to high power efficiency, a complementary cross-coupled Class-C VCO based on the design in [29] is chosen to be implemented. As shown in Figure 4, the NMOS cross-coupling pair $M_{1}$/$M_{2}$ operates in the Class-C scheme. The current mirror including $M_{1}$/$M_{2}$ and $M_{B1}$/$M_{B2}$ work as a start-up circuit. When circuits are powered up, the bias voltage $V_{B}$ is set high enough to ensure the pair $M_{1}$/$M_{2}$ (operating in Class-B) has a enough negative resistance to start to oscillate. Then, currents flowing through $M_{1}$/$M_{2}$ increase, as the oscillating amplitude increases. Thus, currents in $M_{B1}$/$M_{B2}$ increase due to the current mirror scheme. The excess current, flowing in the tail resistor $R_{tail}$, results in lower voltage at $V_{B}$, then forcing the pair $M_{1}$/$M_{2}$ to work in the Class-C domain.

**Figure 5:**
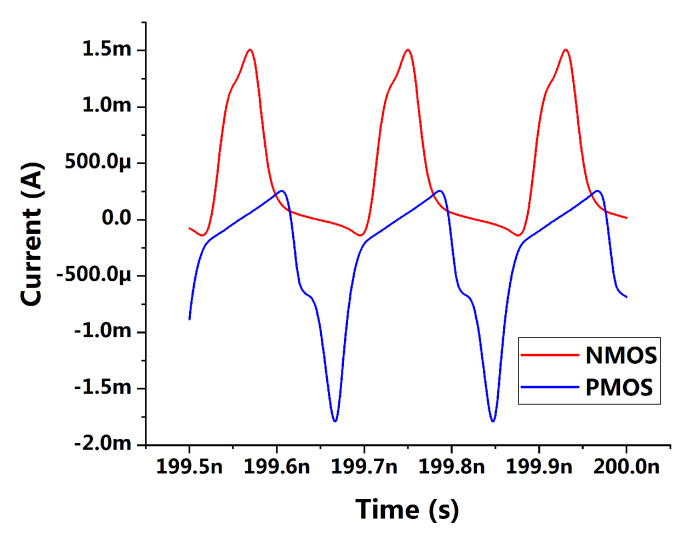
Current-shaping in the Class-C region.

Different from [29], the current source is replaced by a tail resistor in the proposed VCO to eliminate the flicker noise generating from the tail transistor. Moreover, the voltage biasing scheme of the PMOS pairs $M_{3}$/$M_{4}$ is removed, since the DC gain of a operational amplifier (op-amp) could be limited seriously with low supply voltage. The removal of this function would not have much influence on the Class-C operation, since the currents are reused in the pair $M_{1}$/$M_{2}$ and $M_{3}$/$M_{4}$, and Class-C shaping of the current can flow in both $M_{1}$/$M_{2}$ and $M_{3}$/$M_{4}$ as well. To verify that, the current shapings are shown in Figure 5 with transient simulation.

**Figure 6:**
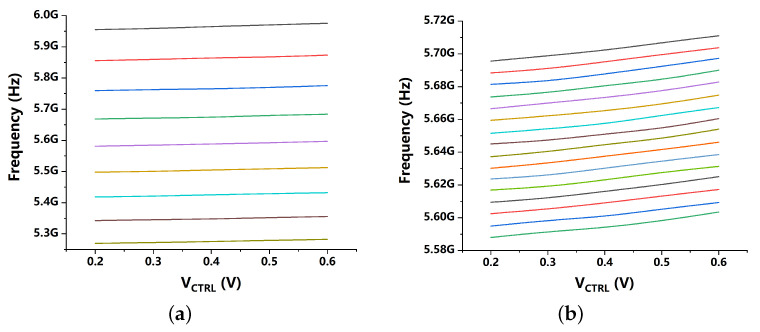
Tuning curves of the proposed VCO. (**a**) coarse-tuning curves, (**b**) fine-tuning curves.

Moreover, the sub-sampling loop of the PLL can lock at an arbitrary integer-N ratio of the reference frequency $f_{REF}$ without an FLL [22], thus, the smallest tuning step of capacitor banks should smaller than $f_{REF}$ to allow ADFLL to select proper digital-control bits for the VCO. In order to ensure the desired large frequency tuning range (about 10%) as well, a 16-bit fine cap bank and an 8-bit coarse cap bank are proposed (Figure 4). Figure 6 captures the both tuning curves of the coarse bank and the fine bank.

### 3.2. Voltage-Controlled Buffer

A VCO buffer is necessary to isolate the VCO and the MSSF, because the sampling capacitor changes the load of the VCO during a complete sampling period if the MSSF is directly connected with outputs of the VCO, which causes considerable spur noise.

**Figure 7:**
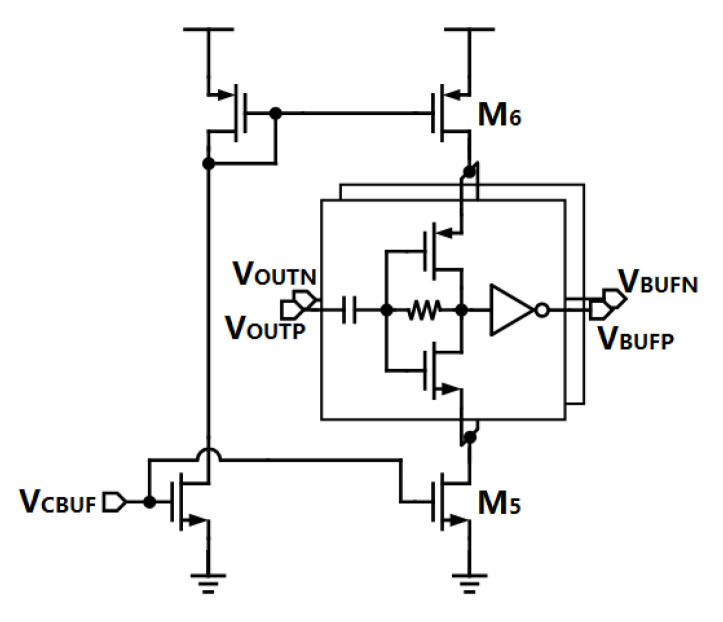
Structure of the proposed VCO buffer.

As in Equation (8), $f_{BW}$ of the proposed SSPLL is determined by the P-path, thus, the bandwidth of the system cannot be adjusted by the pulser as in the conventional SSPLL. The only parameter left to play with in Equation (8) is the amplitude of the VCO buffer. The proposed structure is shown in Figure 7. The input voltage $V_{CBUF}$ controls the current source $M_{5}$ and $M_{6}$ to change the drive capability of the self-biased inverters so as to adjust the output amplitude.

**Figure 8:**
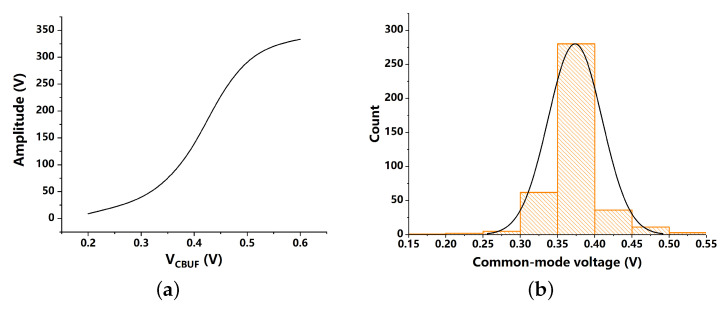
Transient simulation results of the proposed VCO buffer. (**a**) output amplitude variation, (**b**) output common-mode voltage variation.

A series of transient simulations are carried out. Results capture the curve indicating the varying amplitudes as $V_{CBUF}$ increases, as shown in Figure 8a. A Monte-Carlo simulation is carried out in Figure 8b to show the variation of the output common-mode voltages as the process changes.

### 3.3. Rail-to-Rail Sub-Sampling Charge Pump

**Figure 9:**
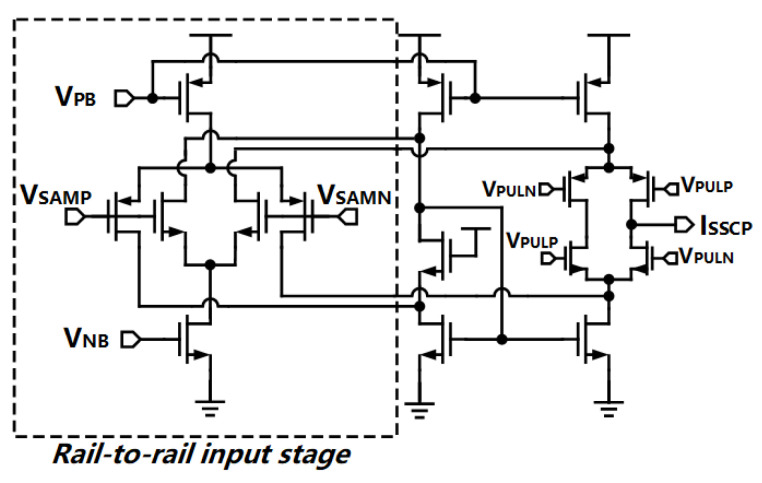
Structure of the proposed rail-to-rail SSCP.

According to the Monte-Carlo simulation results of the VCO buffer, it is noticed that the common-mode voltages have a large range changing from 0.15 V to 0.55 V which may let the input transistors of the traditional SSCP be switched off. In order to mitigate this serious problem, we propose a rail-to-rail SSCP as shown in Figure 9.

**Figure 10:**
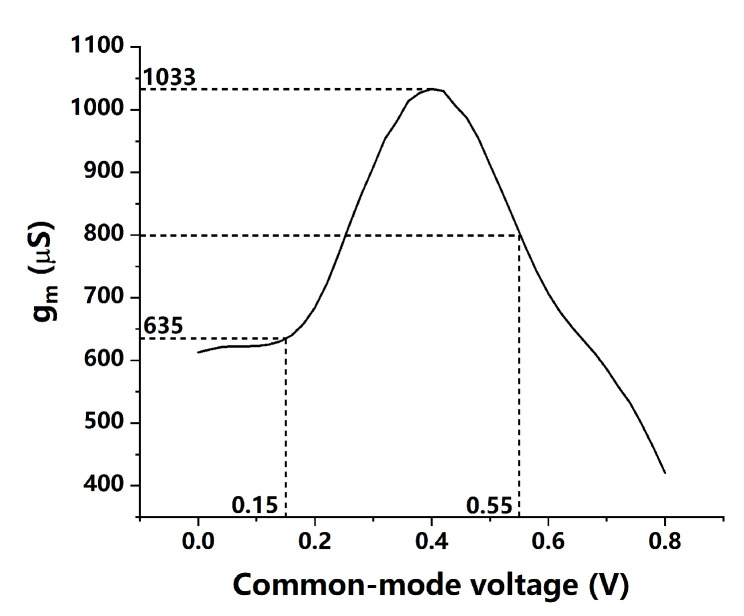
Variation of transconductor of the SSCP as input common-mode voltage changes.

The rail-to-rail input stage is adopted by the folded structure and the headrooms of the current mirror transistors are relieved, which is suitable for the low voltage design. The transconductor $g_{m}$ is simulated as the input common-mode voltage varies as shown in Figure 10. The complementary inputs of the SSCP ensure that at least one pair of transistors (NMOS or PMOS) are always be turned on. The value of $g_{m}$ shows 635 μS and 800 μS as the output common-mode voltage of the VCO buffer appears at the worst cases of the variation. Although the variation of $g_{m}$ is 38.5%, the noise contribution of the proposed SSCP is quite small, thus, this variation can be neglected (we will discuss this in the following section).

## 4. Phase Noise Analysis

**Figure 11:**
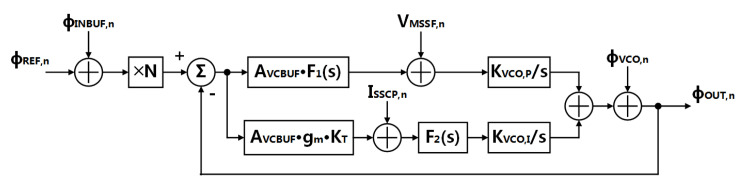
Phase noise linear model of the proposed SSPLL.

An s-domain phase noise model is shown in Figure 11. The noise contribution of the LPF is neglected.

**Figure 12:**
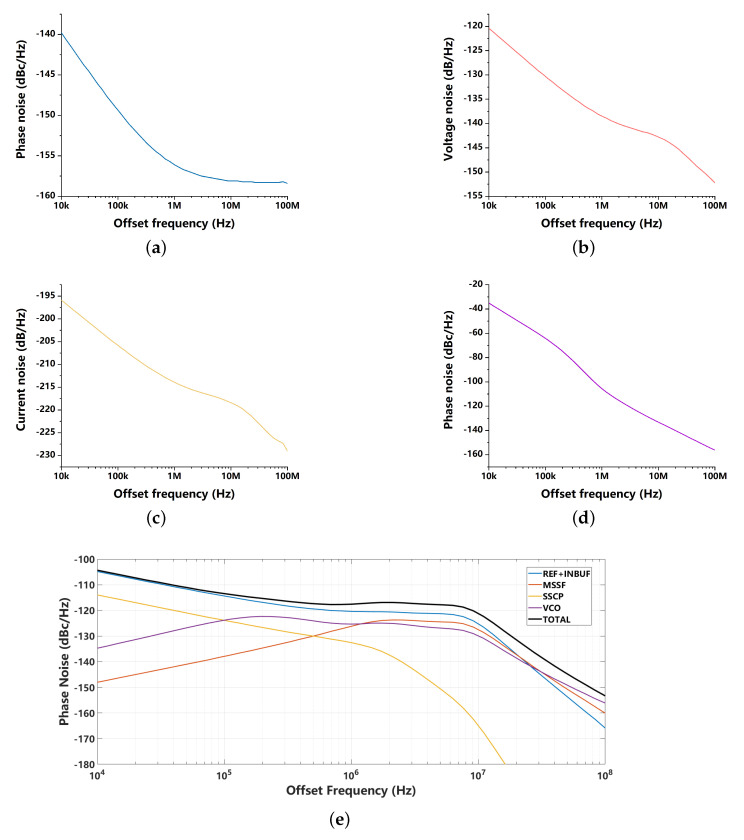
Fitted phase noise: (**a**) phase noise of the reference clock and the input buffer; (**b**) voltage noise of the MSSF; (**c**) current noise of the SSCP; (**d**) phase noise of the VCO; (**e**) phase noise of the SSPLL.

According to transfer function of each part, the whole system phase noise is given as:

(9) $$\begin{array}{c} \phi_{OUT,n}^{2}=\left(\phi_{REF,n}^{2}+\phi_{INBUF,n}^{2}\right)\cdot {\left|\frac{N\cdot G_{SSPLL}\left(s\right)}{1+G_{SSPLL}\left(s\right)}\right|}^{2}+V_{MSSF,n}^{2}\cdot {\left|\frac{1}{1+G_{SSPLL}\left(s\right)}\cdot \frac{K_{VCO}}{s}\right|}^{2}\\ +I_{SSCP,n}^{2}\cdot {\left|\frac{1}{1+G_{SSPLL}}\cdot \frac{K_{VCO}}{s^{2}C_{int1}}\right|}^{2}+\phi_{VCO,n}^{2}\cdot {\left|\frac{1}{1+G_{SSPLL}\left(s\right)}\right|}^{2} \end{array}$$

where $\phi_{REF,n}$, $\phi_{INBUF,n}$ and $\phi_{VCO,n}$ are the phase noise contribution from the reference clock, the input buffer and the VCO, respectively, $V_{MSSF,n}$ is the voltage noise from the MSSF, $I_{SSCP,n}$ is the current noise from the SSCP. Combining with the simulated noise from each part, the fitted phase noise at the output of the SSPLL system is shown in Figure 12. We find that the noise contribution from the SSCP (I-path) is much smaller than the noise from the MSSF (P-path), since the I-path gain $G_{IP}\left(s\right)$ is much smaller than the P-path gain $G_{PP}\left(s\right)$. Thus, compared with traditional SSPLL, the degraded noise feature of the SSCP cannot have much influence on the noise performance of the proposed dual path SSPLL. The calculated integrated RMS jitter in Figure 12 is 180 fs.

## 5. Measurement Results

**Figure 13:**
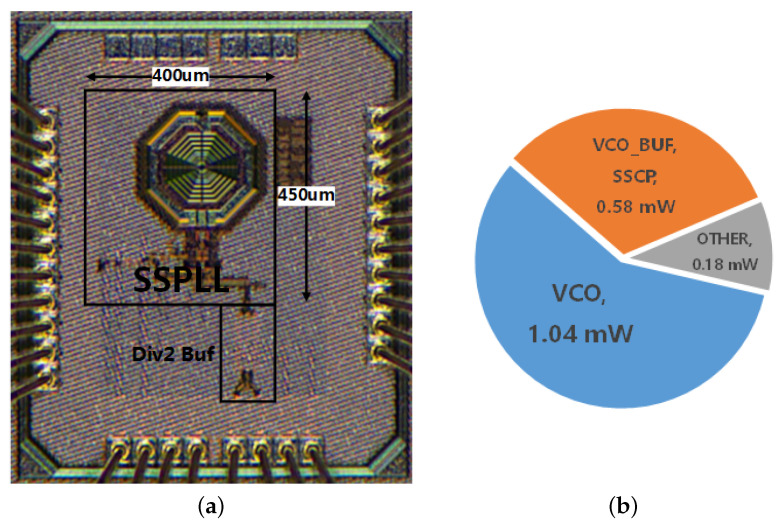
(**a**) chip micro-photo and (**b**) power dissipation breakdown.

The proposed low-voltage hybrid dual-path SSPLL is fabricated in 40-nm CMOS technology. The chip micrograph is shown in Figure 13 and the active core area is 450 × 400 μm${}^{2}$. The power dissipation breakdown is also captured in Figure 13.

**Figure 14:**
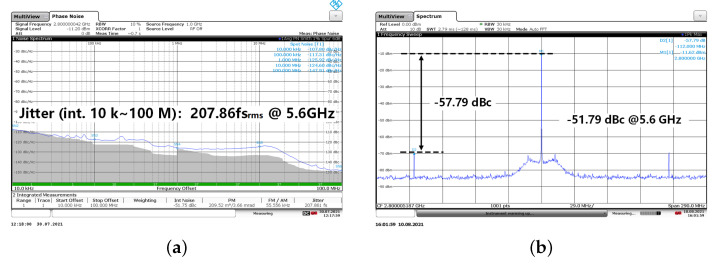
(**a**) phase noise and (**b**) spur noise at central output frequency (divided by 2).

A Rohde & Schwarz FSWP50 phase noise analyzer and a Rohde & Schwarz FSW50 spectrum analyzer are used to test the phase noise and spur noise of the proposed SSPLL, respectively. The measured output frequency range is 5.3 to 5.9 GHz, thus, the central output frequency is 5.6 GHz. Figure 14 shows the phase noise and spur at the central frequency (divided by 2, at 2.8 GHz). The tested RMS integrated phase noise is 207.9 fs. The in-band phase noise at 1 MHz offset frequency is −125.9 dBc/Hz which is −119.9 dBc/Hz when the output frequency is referred at 5.6 GHz. Moreover, the tested spur is −51.8 dBc at central frequency as shown in Figure 14b.

**Figure 15:**
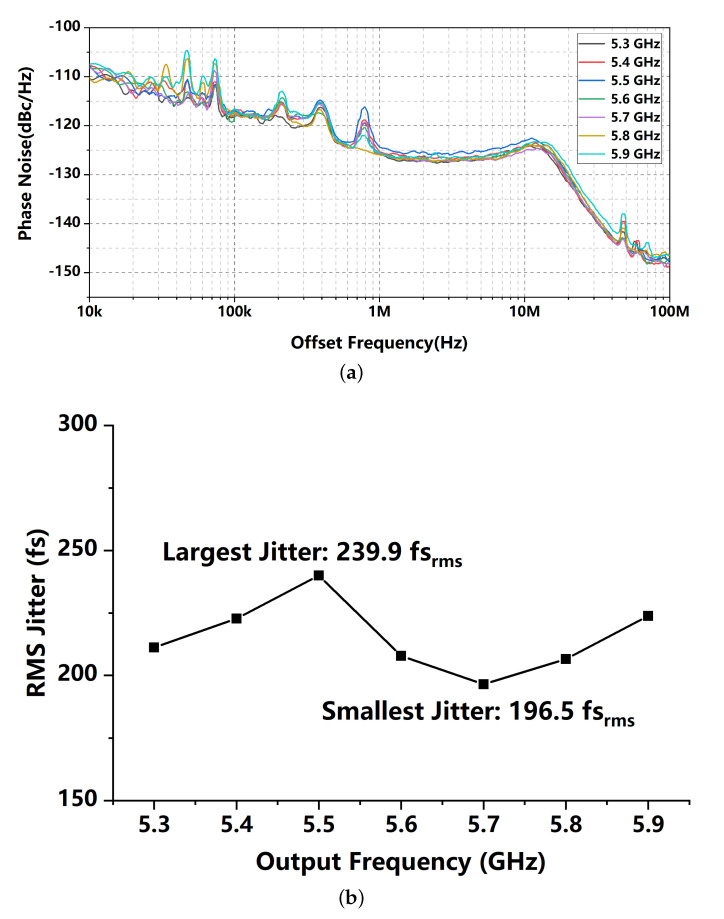
(**a**) phase noise curves and (**b**) integrated jitters covering the frequency range from 5.3 GHz to 5.9 GHz.

Figure 15 shows the curves of tested phase noise covering the output frequency range from 5.3 GHz to 5.9 GHz. The largest integrated jitter appears at 5.5 GHz and the smallest integrated jitter shows at 5.7 GHz. Combining with the power consumption, the worst and the best FoM (from Equation (1)) of the proposed circuit are −249.8 dB and −251.6 dB, respectively.

Table 1 shows the results of this study compared with the results of prior art PLLs. The proposed SSPLL achieves good phase noise performance while achieving low power dissipation.

## 6. Conclusions

This paper proposes a hybrid dual path SSPLL, including a P-path and an I-path, with 0.8 V supply voltage which is fabricated in the 40-nm CMOS technology. A differential MSSF, replacing the SSCP, composed the P-path to avoid the degraded feature caused by the decreasing of the supply voltage. The I-path is built by a rail-to-rail SSCP to suppress the phase noise of the VCO and avoid the trouble of locking at the non-zero phase offset (as in type-I PLL). The measurement results indicate that the SSPLL can operate at the 5.3 to 5.9 GHz frequency range, 196.5 fs RMS integrated jitter and 1.8 mW power dissipation with −251.6 dB FoM. Compared with prior art PLLs, this SSPLL reaches a good phase noise performance with low power dissipation.

## Figures and Tables

**Table 1:** Performance comparison.

	Tech. (nm)	Supply (V)	Out Freq. (GHz)	Ref. Freq. (MHz)	Ref. Spur (dBc)	RMS Jitter (fs)	Power (mW)	FoM (dB)
**This work**	**40**	**0.8**	**5.6**	**112**	**−51.8**	**196.5 (10 k–100 M)**	**1.8**	**−251.6**
[6]	65	0.9	6.8	106.25	−40	190 (10 k–100 M)	2.25	−251
[8]	65	0.45	2.4	10	−50.1	2800 (1 k–100 M)	0.265	−236.8
[15]	28	-	7.77	80	−66.4	82 (10 k–10 M)	14.7	−250
[23]	16	0.9/1.8	18	450	-	164 (1 k–10 M)	29.2	−241
[24]	65	1.2	5	50	−77	357 (10 k–10 M)	3.9	−243
